# Responses of cotton jassid (*Amrasca biguttula*) to synthetic insecticides recommended in Tanzania

**DOI:** 10.3389/finsc.2026.1774983

**Published:** 2026-04-20

**Authors:** Joseph Elias Nyesse, Gration Mutashoberwa Rwegasira, Luseko Amos Chilagane

**Affiliations:** 1 Depertment of Regulatory Services, Tanzania Cotton Board (TCB), Mwanza, Tanzania; 2 Department of Crop Science and Horticulture, Sokoine University of Agriculture, Morogoro, Tanzania

**Keywords:** cotton, cotton crop, effectiveness, insecticides, jassids

## Abstract

**Introduction:**

Cotton jassids (*Amrasca biguttula*) have emerged as the most damaging sucking pest in cotton production in Tanzania, causing yield losses of up to 50% when unmanaged. Control has largely depended on synthetic insecticides; however, their effectiveness has been questioned by growers. It remains unclear whether this reduced efficacy is due to inherent properties of the insecticides or to external factors such as application rate, timing, and method.

**Methods:**

Replicated field experiments were conducted at three locations within the Western and Eastern Cotton Growing Areas (WCGA and ECGA) of Tanzania during the 2024–2025 growing season. Four commonly recommended insecticides: Lambda-cyhalothrin, Chlorpyrifos, Imidacloprid, and Profenofos were evaluated alongside an untreated control using a randomised complete block design (RCBD). Applications were based on the economic threshold level (ETL = 1–2 jassids per plant) at 10 –14 day intervals. Mortality was assessed at 24, 48, and 72 hours post-application, and population reduction was calculated using Abbott’s formula. Data were analysed using analysis of variance (ANOVA), with means separated using Duncan’s Multiple Range Test (DMRT) at 95% confidence.

**Results:**

All tested insecticides significantly reduced both nymph and adult jassid populations compared to the control, although their efficacy varied. Imidacloprid was the most effective, achieving mean reductions of 67.59% (nymphs) and 66.31% (adults), followed by profenofos with reductions of 52.75% (nymphs) and 55.83% (adults).

**Discussion:**

The results demonstrate that insecticide effectiveness varies considerably, with Imidacloprid showing superior performance under field conditions. These findings suggest that its inclusion in integrated pest management (IPM) programmes could improve jassid control. However, optimisation of application practices remains essential to enhance overall efficacy and sustainability.

## 1 Introduction

Cotton (*Gossypium hirsutum L.*) is a vital fiber crop, cultivated commercially in over 50 countries worldwide (1). In Tanzania, it is cultivated in 17 regions, providing employment and income for over 500,000 households (2). It makes a substantial contribution to export revenues and employment, particularly in the Western Cotton Growing Area (WCGA) and the Eastern Cotton Growing Area (ECGA) (3). The WCGA is composed of administrative regions such as Shinyanga, Simiyu, Mwanza, Mara, Geita, Tabora, Kigoma, Katavi, Kagera, Singida, Manyara, and parts of Dodoma and accounts for 97%–99% of total cotton production. The ECGA encompasses regions such as Morogoro, Coast, Kilimanjaro, Iringa, and Tanga, which together account for 1%–3% of gross cotton production (3, 4). Data from the past 5 years indicate that cotton contributed to the nation’s gross domestic product (GDP), generating earnings of USD 243.07 million in 2019/20, USD 79.25 million in 2020/21, USD 125.79 million in 2021/22, USD 237.38 million in 2022/23, USD 276.82 million in 2023/24, and USD 117.23 million in 2024/25 (5). Worldwide, cotton production has for a long time been affected by several factors, including climate change; diseases; insect pests; low and improper use of inputs like pesticides, fertilizers, and seeds; and limited labor availability, transportation of inputs, and market pricing (6). Insect pests and diseases can significantly impact on cotton crops, leading to yield losses if not effectively managed.

Worldwide, the cotton plant hosts approximately 1,300 insect pest species that cause crop damage, resulting in potential yield losses exceeding 80% without crop protection measures and actual losses of 26%–29% even with current management (7, 8). In developing countries of Asia and Africa, insect pest infestations alone can reduce cotton yields by 50% or more (3, 9–13). Bollworms, such as the African bollworm (*Helicoverpa armigera*) and the fall armyworm (*Spodoptera frugiperda*), are major chewing insect pests of cotton. Larvae of these pests feed on leaves, buds, squares, and flowers and damage bolls, which result in yield losses ranging from 10% to 30%. Cotton jassids are devastating sucking pests in cotton-growing areas of Tanzania with a negative impact on the length and strength of cotton lint by feeding on sap from tender plant parts. This reduces plant vigor and affects lint development, leading to significant yield losses if not controlled (4, 14–16).

In *Amrasca biguttula*, eggs hatch within 6–10 days (mean 6.4–7.5 days at 27 °C–34 °C and 60%–70% RH), and the nymphal period lasts 7–21 days depending on the host plant and temperature. The complete egg-to-adult development cycle takes 15–46 days depending on environmental conditions (17–19). The species completes 7–11 overlapping generations per year in tropical climates (20, 21). They suck sap from plant cells and inject phytotoxic saliva that inhibits photosynthesis, causing characteristic “hopper burn” (phytotoxemia) symptoms (22, 23). This leads to cupping, and leaf drop, resulting in stunted plant growth and significantly reduced cotton productivity (24, 25). Although *A. biguttula* has been associated with certain phytoplasma species, its status as a confirmed virus vector has not been established (18). Jassid infestations on cotton begin early in the crop season and persist throughout, with the highest population recorded during the vegetative phase due to the succulence of plant tissues (26). Environmental factors such as temperature, humidity, and rainfall play a crucial role in jassid population dynamics (20). Warm temperatures and moderate humidity levels favor rapid reproduction, whereas heavy rainfall suppresses their population by washing away nymphs and adults. Additionally, dry conditions can enhance jassid survival and feeding activity, leading to greater crop damage (11, 27).

In recent years, cotton jassids (*Amrasca* spp.) have been reported in many countries around the world causing serious damages to cotton crops, including Côte d’Ivoire, Burkina Faso, Cameroon, Niger, Benin, India, Pakistan, Thailand, and Bangladesh (24, 26, 28–31). Other major sucking pests include thrips (*Thrips tabaci*), the cotton aphid (*Aphis gossypii*), the cotton mealybug (*Phenacoccus solenopsis*), and *Dysdercus* spp. Cotton jassids were rarely reported in Tanzania from the 1960s till recent years (32), which suggested that the pests could have been eradicated. The cotton jassid species present in Tanzania has been identified as *A. biguttula*, a polyphagous pest of Asian origin that has recently invaded several African countries (26, 33, 34). In West Africa, *A. biguttula* rapidly displaced the native *Jacobiasca lybica* as the dominant jassid species in cotton fields within a single season (26). Climate suitability modeling has identified Tanzania as highly threatened by *A. biguttula* invasion (34). Although the present study did not include molecular confirmation of species identity, the field specimens were morphologically consistent with *A. biguttula* based on published descriptions (35).

The ICAC (4) reported that jassids are aggressive and the most destructive pests in cotton production in the WCGA. This has led to changes in insecticide spray regimes, increasing the number of sprays from three or four times to four or eight times, as farmers strive to overcome damages mainly inflicted by the pest on cotton in both the ECGA and WCGA (3, 4). The increased number of insecticide sprays has increased the demand for insecticide leading to a steady increase in the amount of insecticides supplied to farmers (5). Consequently, the cotton production costs have skyrocketed in Tanzania, making the crop less profitable to growers. Although the government continues to subsidize insecticides and sprayers to help offset these costs, overall cotton yields have continued to decline due to persistent pest pressure. According to Singh et al. (2), the increased number of insecticide sprays puts the farm profits, goodwill, and country revenue at stake. The efforts put up by the Tanzania Government through the Cotton Board (TCB) in recent years have somewhat paid off, resulting in increased crop productivity over the past 5 years (5). Arguably, the observed increase in cotton production is not much to celebrate, as it is due to excessive investment in insecticide application.

The rationale of increasing insecticide application regimes up to eight times on a crop per season not only is non-profitable but also puts the country’s environment and lives at stake, including farmers, pollinators, and the entire ecosystem. There is also a risk of the target pests developing resistance to the applied insecticides. Sucking pests, particularly jassids, have been reported as the most difficult to control due to their small size, high reproductive potential, and cryptic feeding behavior on the underside of leaves (36, 37), necessitating repeated insecticide applications. Whether the problem is related to the limited responsiveness of the jassids to the used insecticides, poor application techniques, or short life circles and low threshold of the jassid population (economic threshold level = 1–2 jassids per plant) that necessitates short repeats after every new generation has not been explored. Considering all of these factors, the current study was conducted to investigate the responsiveness of cotton jassids to the different insecticides recommended for use under field conditions. The study aimed to evaluate the comparative efficacy of four recommended synthetic insecticides against cotton jassids under field conditions across different cotton-growing areas of Tanzania, in order to inform evidence-based pest management decisions.

## 2 Materials and methods

### 2.1 Study area and experimental design

**Figure 1:**
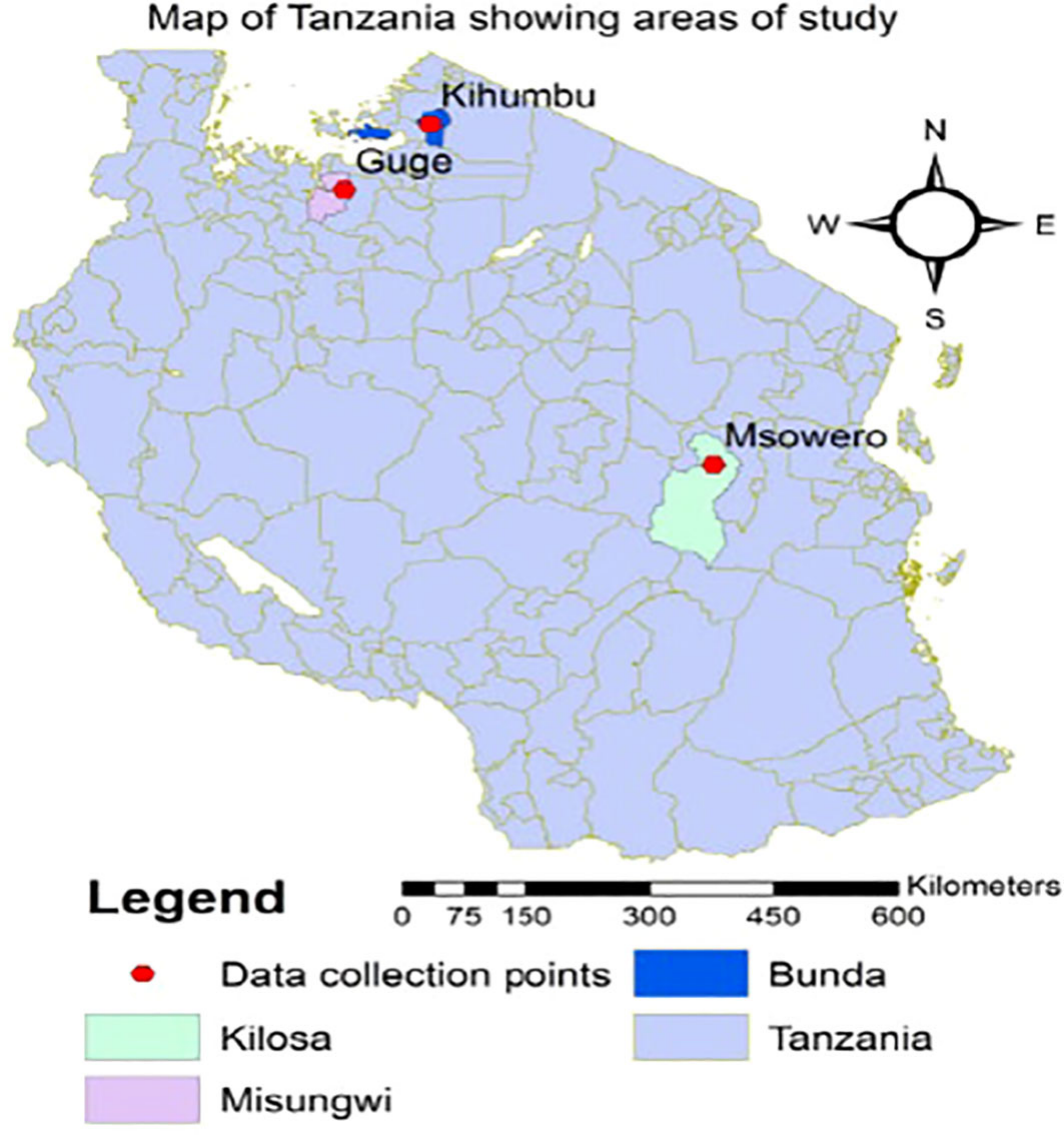
Map of Tanzania highlighting experimental sites, with Bunda in blue, Kilosa in green, and Misungwi in purple. Data collection points are marked with red hexagons at Kihumbu, Guge, and Msowero. A north arrow and distance scale are included.

**Figure 2:**
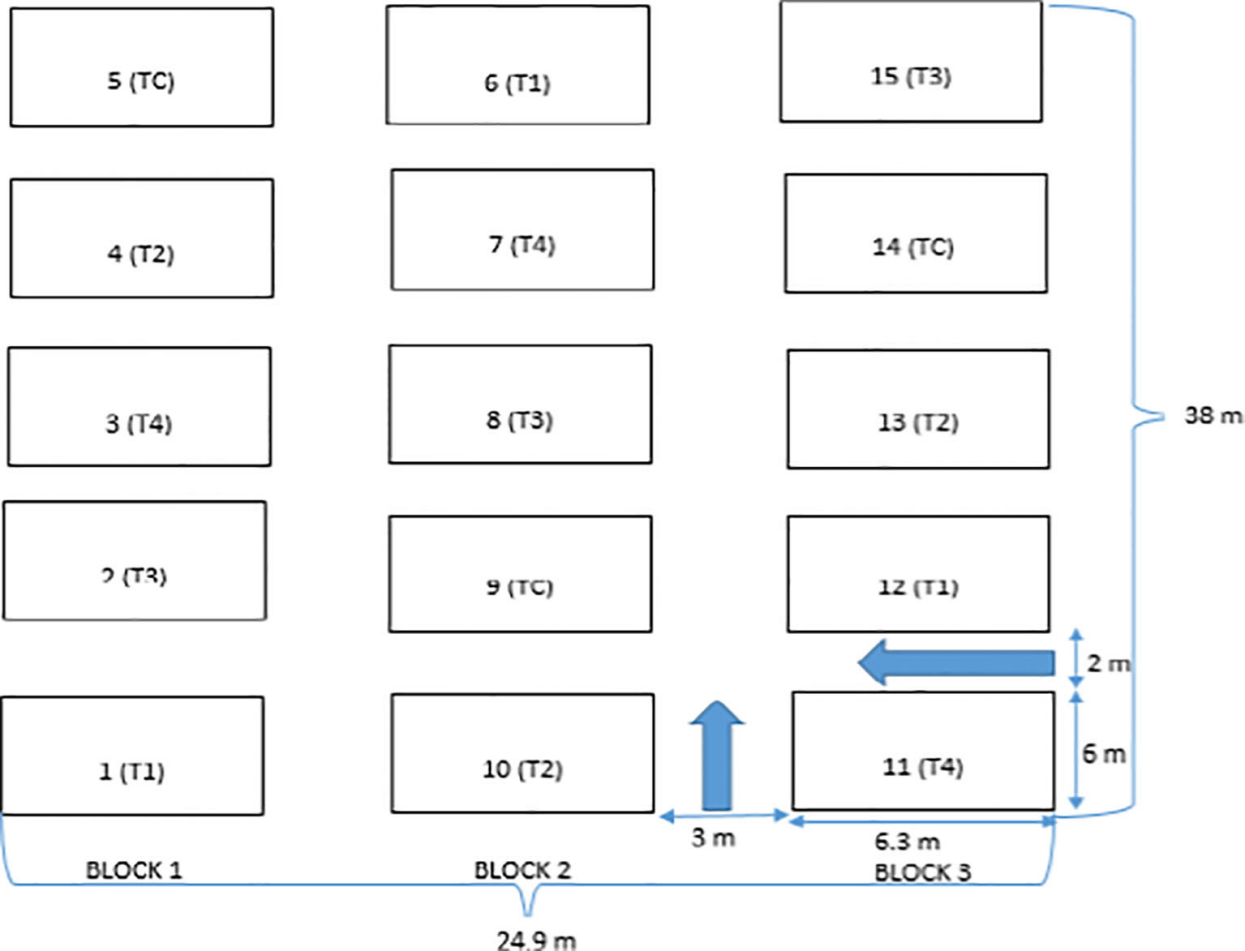
The figure shows a schematic layout of a Randomised Complete Block Design (RCBD) used in a cotton field trial conducted at Misungwi, Bunda, and Kilosa sites. The experimental area is rectangular, measuring 24.9 m in width and 38 m in length. The design consists of multiple blocks separated by 3 m spacing. Each block contains uniformly sized plots measuring 6.3 m by 6 m. Individual plots are labelled with treatment codes: T1 (imidacloprid), T2 (lambda-cyhalothrin), T3 (chlorpyrifos), T4 (profenofos), and TC (untreated control). Each numbered square represents a treatment plot arranged in a randomized pattern within blocks for field experimentation.

The field experiments were carried out during the 2024/2025 cropping season in three districts in Tanzania with varied conditions, namely, Misungwi (2°51'3.6" S, 33°10'2.6" E) and Bunda (1°58'51.3" S, 34°5'51.4" E), which are located in the WCGA, and Kilosa (6°50'55.4" S, 37°39'30.8" E), which is located in the ECGA (**Figure 1**). The commercial cotton variety UKM08 was planted on 14 November 2024 in Misungwi, 16 November 2024 in Bunda, and 23 January 2025 in Kilosa. Initial insecticide applications were initiated when cotton jassid populations reached the economic threshold level (ETL) of one to two jassids per plant. Spraying commenced on 20 February 2025 in Misungwi, 27 February 2025 in Bunda, and 21 February 2025 in Kilosa, with subsequent applications carried out at 10–14 day intervals at each site. The experiment was set up using a randomized complete block design (RCBD) (**Figure 2**) with three replications and five treatments: Imidacloprid, Lambda-cyhalothrin, Chlorpyrifos, Profenofos, and an untreated negative control (**Table 1**). All insecticides were applied at the recommended dosage (**Table 1**) using knapsack sprayers. Each plot measured 6.0 m by 6.3 m (34.2 m²), with plant spacing maintained at 30 cm within rows and 60 cm between rows. Standard agronomic practices were consistently applied across all treatments during the trial. These included ox-ploughing for land preparation, thinning at 3 weeks after emergence, manual weeding starting 3 weeks post-emergence, and NPK (20:10:5) fertilizer at 50 kg/ha applied at planting.

**Table 1:** Insecticides tested against cotton jassids, including active ingredients, chemical classes, and trade names, modes of action, target orders, and application dosages.

SN.	Active ingredient	Chemical class	Trade name	General mode of action	Main target orders	Dosage/Liter of water
1	Imidacloprid	Neonicotinoid	BRAVO 20EC	Systemic (nAChR agonist)	Hemiptera, Thysanoptera	2mL/1.5Lt
2	Lambda-cyhalothrin	Pyrethroid	SAMURAI 5EC	Contact/ingestion (Na+ channel modulator)	Lepidoptera, Hemiptera	2mL/1.5Lt
3	Chlorpyrifos	Organophosphate	DARSFARM 240EC	Contact/ingestion (AChE inhibitor)	Lepidoptera, Coleoptera, Hemiptera	3mL/1.5Lt
4	Profenofos	Organophosphate	MUPAFORCE 720EC	Contact/translaminar (AChE inhibitor)	Lepidoptera, Hemiptera	2mL/1.5Lt

SN, serial number; AChE, acetylcholinesterase; nAChR, nicotinic acetylcholine receptor.

### 2.2 Data collection

Data collection was carried out early in the morning by counting jassids on 10 plants per plot. Each plot contained 210 plants, arranged in 10 rows (spaced 60 cm apart) with 21 plants per row (spaced 30 cm apart). Plants were selected from the central rows (168 plants; 8 rows × 21 plants), excluding one border row on each side to minimize edge effects. A systematic random sampling procedure was applied, with a sampling interval of 14, calculated by dividing the total number of plants in the central rows (168) by 10. Following a random start between plants 1 and 14, every 14th plant was selected and tagged for jassid counting on each sampling round. Both nymph and adult populations were recorded on the top, middle, and bottom leaves of each selected plant. Spraying was initiated when jassids infestation reached the ETL of one to two jassids per plant (38, 39), at intervals of 10 to 14 days. Depending on the manufacturer’s recommendation, insecticide solutions were prepared using volume/volume and weight/volume formulations and applied using a knapsack sprayer equipped with a hollow cone nozzle. Spraying was performed in the morning before 9:00 a.m. Data on live jassid populations (nymphs and adults) were recorded both before and after insecticide application. Pretreatment observations were made immediately prior to spraying, whereas post treatment data were collected at 24, 48, and 72 h following application in accordance with Abbas et al. (40).

### 2.3 Data analysis

The collected data were tested for normality using the Shapiro–Wilk test and thereafter subjected to analysis of variance (ANOVA) using GenStat 12th Edition software. Treatment means were separated using the least significant difference (LSD) test at a 5% significance level according to Irshad et al. (39). Prior to pooling data across sites (**Tables 2a**-**d**), a two-way ANOVA was performed to test for site × treatment interaction effects (**Tables 3a**-**d**). The percentage reduction per plant in cotton jassid populations (hereafter termed “corrected mortality”) was calculated using Abbott’s formula (41; reprinted 1987), which adjusts for natural mortality observed in the untreated control plots: Corrected mortality (%) = [(Population in the control − Population in the treatment)/Population in the control] × 100 where “population” refers to the mean number of living jassid specimens observed per plant. This correction was applied systematically to all treatment data. In the control plots, jassid populations generally increased over time rather than showing mortality, confirming that the formula was appropriate for isolating treatment effects rather than correcting for natural mortality (25). In addition, repeated-measures ANOVA was performed to evaluate treatment effects across multiple spray applications (sprays 1, 2, and 3), with time as the within-subject factor and treatment as the between-subject factor. This approach accounts for the temporal dependency of observations within each experimental unit across successive spray rounds. The results of the repeated-measures ANOVA are presented in **Tables 2a, b**.

**Table 2a:** Evaluation of the insecticide effects on adult cotton jassid life stages over spray applications at Bunda, Kilosa, and Misungwi sites in Tanzania (2024–2025 season).

Repeated-measures ANOVA for adult jassids			
Before spray	Spray 1	Spray 2	Spray 3
19.39	3.89	1.61	9.36
103.99***	416.21***	388.04***	879.19***
243.03***	30.82***	10.94^ns^	30.59**
18.8^ns^	5.93**	4.95^ns^	19.09***
19.76	1.72	3.581	4.66

Repeated-measures ANOVA results for adult cotton jassid populations. sprays 1–3 = post-application assessments. ***, **, * = significant at p < 0.001, 0.01, 0.05; ns = not significant. TRT, treatment (insecticides vs. control); TIME, post-spray intervals (24, 48, 72 h); TRT.TIME, treatment × time interaction; REP, replication; residual, error term.

**Table 2b:** Evaluation of the insecticide effects on nymph cotton jassid life stages over spray applications at Bunda, Kilosa, and Misungwi sites in Tanzania (2024–2025 season).

Repeated-measures ANOVA for nymph jassids				
Source of variation	Before spray	Spray 1	Spray 2	Spray 3
REP	9.16	5.96	13.78	3.941
TRT	96.97**	263.54***	509.46***	906.37***
TIME	116.12*	34.82***	20.50**	18.81**
TRT.TIME	55.86^ns^	10.29***	6.42^ns^	7.56**
RESIDUAL	30.07	1.49	3.41	3.31

Repeated-measures ANOVA results for nymph cotton jassid populations. Spray 1–3 = post-application assessments. ***, **, * = significant at p < 0.001, 0.01, 0.05; ns = not significant. TRT, treatment (insecticides vs. control); TIME, post-spray intervals (24, 48, 72 h); TRT.TIME, treatment × time interaction; REP, replication; residual, error term.

**Table 2c:** Mean percentage reduction of five treatments against adult jassid populations across three sites (Bunda, Kilosa, and Misungwi), Tanzania (2024–2025 season), assessed over three consecutive spray applications.

Adult jassids								
TRT	Before spray	After spray 1	r%-mean	After spray 2	r%-mean	After spray 3	r%-mean	AV - mean
T1	6.52	2.22	65.95	2.11	67.64	2.26	65.34	66.31
T2	9.78	4.7	51.94	5.56	43.15	6.93	29.14	41.41
T3	10.19	6.11	40.04	7.67	24.73	9.26	9.13	24.63
T4	10	3.7	63.00	3.96	60.40	5.59	44.10	55.83
TC	11.93	12.37	−3.69	11.96	−0.25	17.44	−46.19	−16.71
P-value	<.001	<.001		<.001		<.001		
LSD	1.56	0.92		2.14		2.98		
c.v (%)	16.8	16.6		35.9		37.60		

TRT, treatment; T1–T4, insecticide treatments (Imidacloprid, Lambda-cyhalothrin, Chlorpyrifos, Profenofos); TC, untreated control. r%-mean, mean percent reduction per plant relative to pre-spray baseline; AV-mean, mean percent reduction per plant relative to pre-spray after three spray round. Data analyzed by ANOVA; P-values < 0.001 indicate highly significant treatment effects; LSD, least significant difference (p < 0.05); CV, coefficient of variation (%).

**Table 2d:** Mean percentage reduction of five treatments against nymph jassid populations across three sites (Bunda, Kilosa, and Misungwi), Tanzania (2024–2025 season), assessed over three consecutive spray applications.

Nymph jassids								
TRT	Before spray	After spray 1	r%-mean	After spray 2	r%-mean	After spray 3	r%-mean	AV-mean
T1	7.93	2.52	68.22	2.78	64.94	2.41	69.61	67.59
T2	11.04	6.63	39.95	7.22	34.60	10.52	4.71	26.42
T3	10.89	5.22	52.07	5.56	48.94	7.74	28.93	43.31
T4	10.3	4.11	60.10	4.3	58.25	6.19	39.90	52.75
TC	13.22	10.74	18.76	13.96	−5.60	17.89	−35.33	−7.39
P-value	0.002	<.001		<.001		<.001		
LSD	2.38	0.98		2.48		5.81		
c.v (%)	23.4	17.6		38.4		68		

TRT, treatment; T1–T4, insecticide treatments (Imidacloprid, Lambda-cyhalothrin, Chlorpyrifos, Profenofos); TC, untreated control. r%-mean, mean percent reduction per plant relative to pre-spray baseline. AV-mean, mean percent reduction per plant relative to pre-spray after three spray round. Data analyzed by ANOVA; P-values < 0.001 indicate highly significant treatment effects; LSD, least significant difference (p < 0.05); CV, coefficient of variation (%).

**Table 3a:** ANOVA results for site × treatment interaction effects on adult cotton jassid populations per plant across study sites over three spray rounds.

ANOVA for interaction—adult jassids												
Source of variation	Before spray 1	24 h	48 h	72 h	Before spray 2	24 h	48 h	72 h	Before spray 3	24 h	48 h	72 h
REP	2.02	0.16	5.07	0.62	1.87	6.2	8.42	0.56	82.47	26.96	0.47	7.8
SITE	41.16**	16.29***	8.27**	0.82^ns^	115.27***	102.87***	37.49***	32.62***	290.6***	119.02***	126.07***	70.07***
TRT	8.26^ns^	102.06***	129.83***	196.19***	37.56***	91.74***	121.26***	184.94***	95.78***	212.08***	285.69***	419.59***
SITE.TRT	10.74^ns^	4.87**	0.60^ns^	0.32^ns^	3.32^ns^	4.39^ns^	3.91^ns^	4.84^ns^	22.54*	5.83^ns^	9.79^ns^	7.79^ns^
RESIDUAL	5.07	1.7	1.61	1.789	6.89	6.61	2.78	3.56	8.16	7.36	8.47	5.8

ANOVA table showing site × treatment interaction effects on adult cotton jassid populations per plant across pre-pray and post-spray (24, 48, 72 h) intervals for three spray rounds. ***, **, * = significant at p < 0.001, 0.01, 0.05; ns, not significant. SITE, study location (Bunda, Kilosa, Misungwi); TRT, treatment (insecticides vs. control); SITE.TRT, site × treatment interaction; REP, replication. Residual, error term.

**Table 3b:** Site × treatment interaction effects on adult cotton jassid mean populations (AV-MEAN) per plant at 24, 48, and 72 h post-application across three spray rounds in Bunda, Kilosa, and Misungwi, Tanzania.

SITE	AV-MEAN				
	Imidacloprid (T1)	Profenofos (T4)	Lambda-cyhalothrin (T2)	Chlorpyrifos (T3)	Control
Kilosa	2.52	5.04	6.7	9.15	15.3
Bunda	2.15	4.81	5.74	7.3	14.04
Misungwi	1.93	3.41	4.74	6.59	12.44

AV-MEAN after three sprays (72h). Full data: **Supplementary Table 3**. P = 0.02–0.99 across time points; LSD = 2.13–4.8

**Table 3c:** ANOVA results for site × treatment interaction effects on nymph cotton jassid populations per plant across study sites over three spray rounds.

ANOVA for interaction—nymph jassids												
Source of variation	Before spray 1	24 h	48 h	72 h	Before spray 2	24 h	48 h	72 h	Before spray 3	24 h	48 h	72 h
REP	5.27	0.62	3.29	3.20	2.96	10.42	7.62	1.867	11.02	3.49	1.62	4.2
SITE	7.20^ns^	4.82^ns^	13.36**	1.27^ns^	161.16***	69.76**	43.02**	36.07***	919.02***	657.69***	456.16***	437.27***
TRT	8.52^ns^	51.02***	88.00***	145.11***	57.41***	110.14***	199.19***	212.97***	142.76***	220.26***	322.94***	378.28***
SITE.TRT	8.26^ns^	2.57^ns^	2.13^ns^	1.04^ns^	5.96^ns^	6.39^ns^	6.69^ns^	11.48^ns^	17.69^ns^	23.94**	36.54**	34.63***
RESIDUAL	5.15	2.74	1.69	1.01	5.69	10.47	5.12	3.56	12.88	7.13	10.38	4.32

ANOVA table showing site × treatment interaction effects on nymph cotton jassid populations across pre-spray and post-spray (24, 48, 72 h) intervals for three spray rounds. ***, **, * = significant at p < 0.001, 0.01, 0.05; ns, not significant. SITE, study location (Bunda, Kilosa, Misungwi); TRT, treatment (insecticides vs. control); SITE.TRT, site × treatment interaction; REP, replication. Residual, error term.

**Table 3d:** Site × treatment interaction effects on nymph cotton jassid mean populations (AV-MEAN) per plant at 24, 48, and 72 h post-application across three spray rounds in Bunda, Kilosa, and Misungwi, Tanzania.

Site	AV-MEAN				
	Imidacloprid (T1)	Lambda-cyhalothrin (T2)	Chlorpyrifos (T3)	Profenofos (T4)	Control (Tc)
Bunda	3	9.96	7.85	6.37	17.26
Kilosa	2.37	8	5.59	4.33	13.56
Misungwi	2.33	6.41	5.07	3.89	11.78

AV-MEAN after three sprays (72h). Full data/P-values/LSD: **Supplementary Table 4**. Significant interactions at Spray 2/3 72h (P<0.01).

## 3 Results

**Figure 3:**
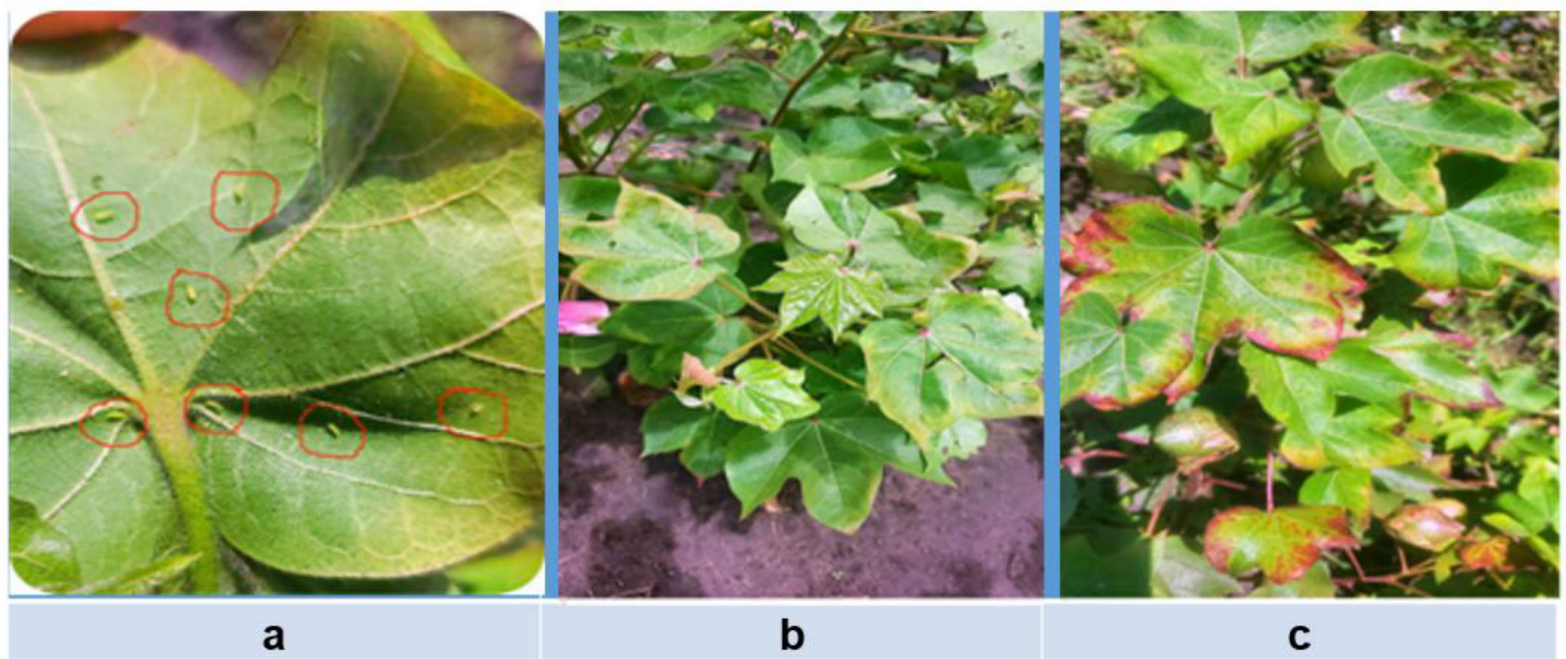
Infestation and symptoms of jassid on cotton crop. **(a)** Jassid infestation on the lower surface of the leaf, **(b)** initial jassid injury symptoms on cotton leaves, and **(c)** advanced jassid injury symptoms on cotton crops. Photos were recorded in Bunda and Misungwi in May 2025.

The populations of cotton jassids recorded before and after insecticide application in the experiments conducted at Kilosa, Misungwi, and Bunda are presented in **Tables 2a–d**, **3a–d**. Jassid infestations cause characteristic damage to cotton plants, including leaf burn, cupping, and premature leaf drop, which ultimately results in stunted plant growth and substantial reductions in cotton productivity (**Figure 3**).

### 3.1 Efficacy of tested insecticides against adult jassids across three spray rounds

**Figure 4:**
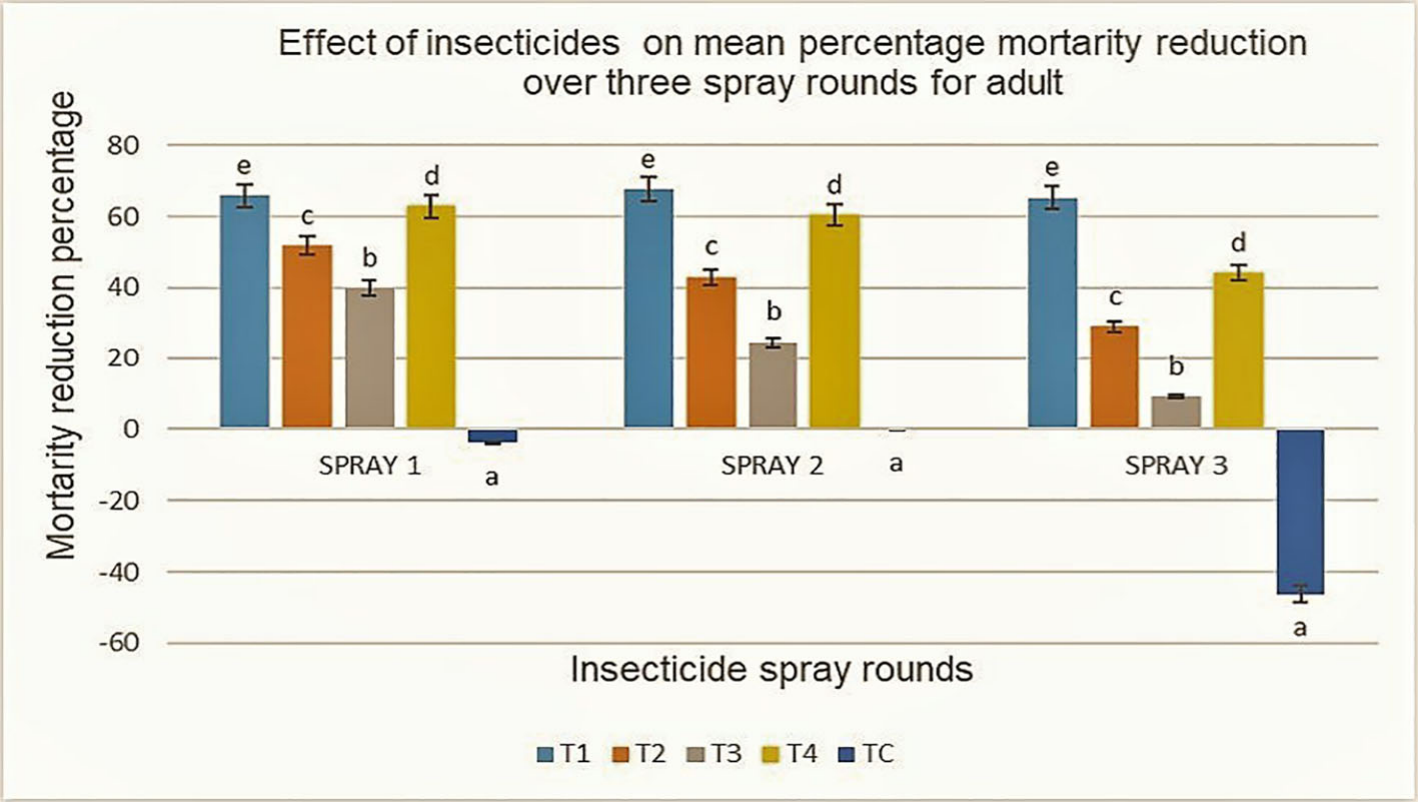
Effect of treatments application on mean percentage mortality reduction for adult insects across three spray rounds with five treatments: T1, T2, T3, T4, and TC. T1 and T4 consistently show the highest reductions, while TC shows negative or zero reduction, including a large negative value after the third spray. Error bars represent variability, and different letters above the bars indicate statistically significant differences.

Most of the tested insecticides were effective in reducing adult jassid populations at different application intervals when compared with the untreated control (**Tables 2a, c**) albeit with varying efficacy. In the first round of spraying, Imidacloprid 20EC demonstrated the highest effectiveness with a 65.95% reduction in jassid population per plant at 72 h after application followed by Profenofos 720EC (63.00%) and Lambda-cyhalothrin 5EC (51.94%). Chlorpyrifos 240EC demonstrated lowest performance with only 40.04% mean jassids’ reduction per plant. Although Chlorpyrifos and Lambda-cyhalothrin were least effective, they still managed to lower jassid populations below the ETL, and their results were statistically significant compared with the control. A similar trend was recorded after the second round of spray in the repeated insecticide application whereby Imidacloprid remained the most effective, reducing jassids by 67.64% after 72 h. Profenofos followed reducing the jassid population by 60.40%, whereas Lambda-cyhalothrin and Chlorpyrifos were relatively less effective with 43.15% and 24.73% reduction in the jassid population, respectively. The third round of spray showed a similar effectiveness whereby Imidacloprid performed the highest at 65.34% reductions after 72 h. Profenofos followed with 44.10% reductions, whereas Lambda-cyhalothrin and Chlorpyrifos remained the least effective, with 29.14% and 9.13%, respectively, across the sites (**Figure 4**).

### 3.2 Efficacy of tested insecticides against nymph jassids across three spray rounds

**Figure 5:**
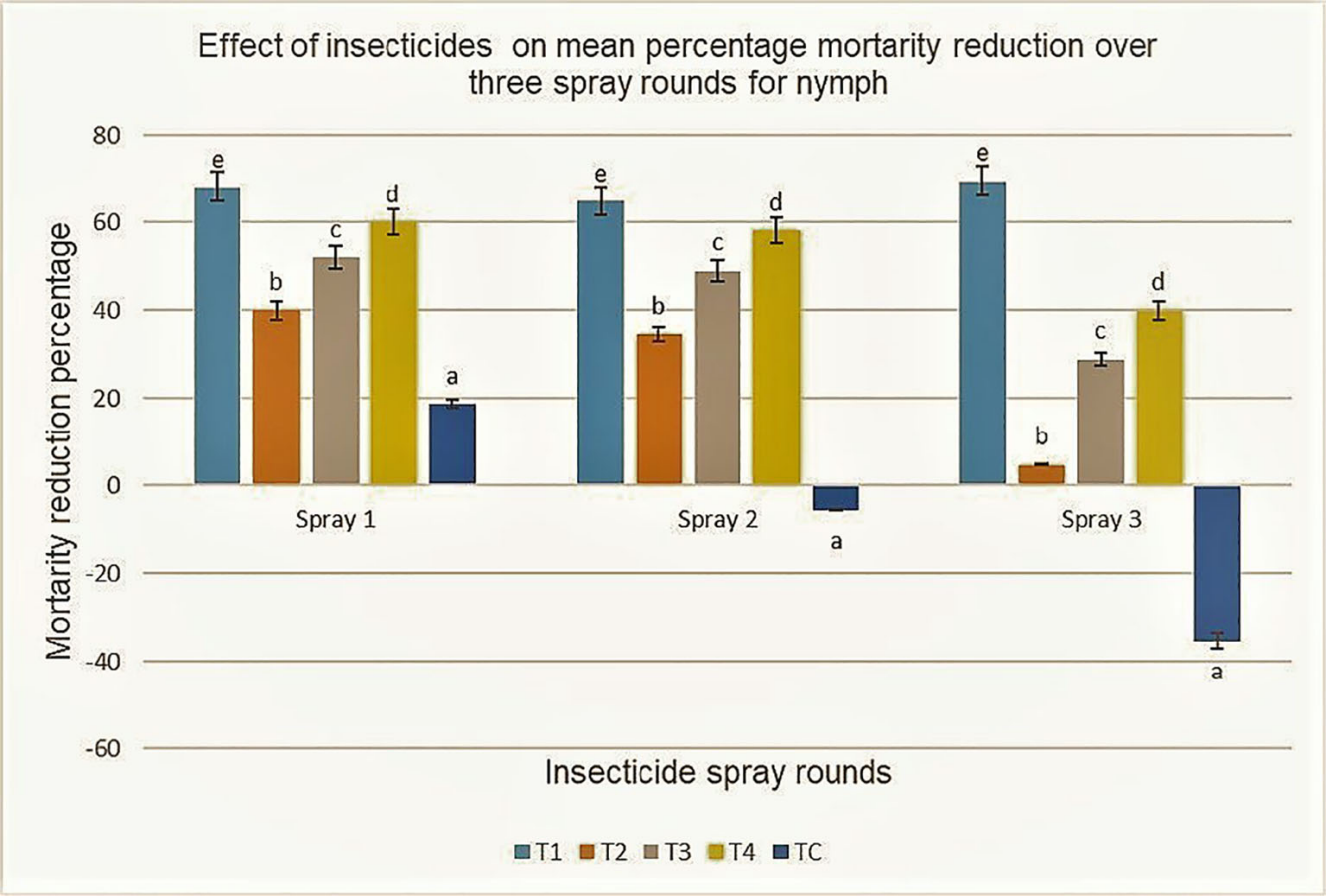
Effect of five insecticide treatments (T1, T2, T3, T4, TC) on mean percentage mortality reduction of nymphs over three spray rounds. T1 consistently produces the highest mortality reduction above 60 percent in all rounds, while TC shows a strong decline to negative values by Spray 3. Error bars are included for each treatment.

The findings demonstrated a significant difference in the mean percentage reduction per plant of nymph jassid populations at 24, 48, and 72 h following the three insecticide applications across the sites using repeated-measures ANOVA (**Tables 2b, d**). Across all three spray rounds, Imidacloprid consistently showed the highest efficacy, followed by Profenofos, whereas Lambda-cyhalothrin and Chlorpyrifos were less effective in comparison. Imidacloprid achieved the greatest reductions in population across all three spray applications, peaking at 68.22% after 72 h in the first spray. Profenofos followed with 60.10%, whereas Chlorpyrifos and Lambda-cyhalothrin were markedly less effective (52.07% and 39.95%, respectively). All treatments caused a significant increase in mortality for up to 72 h after the second application. Imidacloprid (64.94%) and Profenofos (58.25%) remained the most effective, significantly outperforming Chlorpyrifos (48.94%) and Lambda-cyhalothrin (34.60%). A similar trend was observed following the third round of application, with Imidacloprid consistently achieving the highest reduction (69.61%), followed by Profenofos (39.90%). Although Chlorpyrifos (28.93%) was comparatively less effective, it reduced jassid populations below the ETL, whereas Lambda-cyhalothrin was least effective with 4.71% population reduction. Both treatments exhibited statistically significant differences compared with the untreated control in which Imidacloprid and Profenofos were observed to cause more jassids mortality rates than Lambda-cyhalothrin and Chlorpyrifos (**Figure 5**).

### 3.3 Site × treatment interaction effects on adult cotton jassid populations

The interaction between site and treatment indicated variation in the effectiveness of the tested insecticides across the three locations Kilosa, Bunda, and Misungwi (**Tables 3a, b**). Imidacloprid consistently demonstrated the highest effectiveness across all sites, producing the lowest adult jassid populations following insecticide application. The average post-spray populations for Imidacloprid were 2.52, 2.15, and 1.93 adults per plant at Kilosa, Bunda, and Misungwi, respectively, indicating strong and consistent suppression compared with other treatments. At Kilosa, Imidacloprid was the most effective treatment, followed by Profenofos (5.04 adults per plant) and Lambda-cyhalothrin (6.70 adults per plant), whereas Chlorpyrifos recorded relatively higher populations (9.15 adults per plant), suggesting weaker control. Similarly, the untreated control (TC) maintained the highest population (15.30 adults per plant), confirming the effectiveness of the insecticide treatments. At Bunda, Imidacloprid again provided the best control with an average of 2.15 adults per plant after spraying. Profenofos (4.81) and Lambda-cyhalothrin (5.74) provided moderate suppression, whereas Chlorpyrifos (7.30) showed comparatively lower efficacy. The untreated control maintained high populations (14.04 adults per plant). At Misungwi, Imidacloprid also recorded the lowest mean population (1.93 adults per plant), followed by Profenofos (3.41) and Lambda-cyhalothrin (4.74). Chlorpyrifos again showed reduced effectiveness (6.59), whereas the control remained high (12.44). Overall, Imidacloprid consistently performed best across all sites, Profenofos and Lambda-cyhalothrin provided moderate control, and Chlorpyrifos was the least effective among the treated plots, whereas the untreated control recorded the highest jassid populations throughout the study.

### 3.4 Site × treatment interaction effects on cotton jassid nymph populations

The interaction between site and treatment showed noticeable differences in the control of cotton jassid nymphs across Bunda, Kilosa, and Misungwi (**Tables 3c, d**). Imidacloprid consistently performed best at all sites, resulting in the lowest mean nymph populations after spraying compared with other treatments. At Kilosa, Imidacloprid provided the strongest suppression with the lowest average population (2.37 nymphs per plant), followed by Profenofos (4.33) and Chlorpyrifos (5.59), whereas Lambda-cyhalothrin (8.00) showed weaker control. The untreated control (TC) maintained a high population (13.56), indicating continued infestation in the absence of insecticide treatment. At Misungwi, Imidacloprid again recorded the lowest mean population (2.33 nymphs per plant), closely followed by Profenofos (3.89). Chlorpyrifos (5.07) and Lambda-cyhalothrin (6.41) provided moderate suppression but were less effective than Imidacloprid and Profenofos. The untreated control remained high with 11.78 nymphs per plant, demonstrating the persistence of the pest without treatment. At Bunda, Imidacloprid also showed the highest effectiveness with an average of 3.00 nymphs per plant after spraying. Profenofos (6.37) performed moderately well, followed by Chlorpyrifos (7.85), whereas Lambda-cyhalothrin (9.96) recorded relatively higher populations among treated plots. The control plots recorded the highest infestation (17.26 nymphs per plant).

Significant differences among sites were observed, although not consistently across all observation periods. For adult jassids, the main effect of site was significant at most sampling intervals (P ≤ 0.05), except at 72 h after the first spray where no significant differences were detected (**Table 3a**). In contrast, for nymph jassids, site effects were not significant before spraying and during early observations (24 and 72 h after the first spray; P > 0.05) but became significant at 48 h after the first spray (P ≤ 0.01) and remained highly significant throughout the second and third spray rounds (P ≤ 0.001) (**Table 3c**). These results indicate that site-specific differences in jassid populations became more pronounced over time, particularly after repeated insecticide applications. Overall, Imidacloprid was the most effective treatment across all sites, consistently reducing nymph populations to the lowest levels. Profenofos and Chlorpyrifos showed moderate control, whereas Lambda-cyhalothrin was comparatively less effective, and the untreated control consistently maintained the highest nymph populations.

## 4 Discussion

This field study evaluated the response of cotton jassids (both nymphs and adults) on different synthetic insecticides with active ingredients (Imidacloprid, Profenofos, Lambda-cyhalothrin, and Chlorpyrifos) including untreated control during the 2024/2025 cropping season. It has provided promising preliminary evidence on the comparative efficacy of selected insecticides against cotton jassids under experimental conditions. It was established that across all sites and at all three assessment intervals (24, 48, and 72 h after application), Imidacloprid-based insecticide exhibited highest efficacy against both adult and nymph jassids after 72 h, followed by Profenofos-based insecticide. Site-specific results showed that Imidacloprid was more effective in controlling nymph jassids at Misungwi and Kilosa than at Bunda. In contrast, for adult jassids, Imidacloprid performed better at Misungwi and Bunda compared with Kilosa. These findings suggest differences in pest susceptibility and suggest that environmental conditions, such as differences in temperature, rainfall patterns, and relative humidity between the WCGA and ECGA sites, may influence insecticide performance (42, 43). Although Chlorpyrifos-based insecticide scored third position, it was more effective at reducing the population of nymphs rather than adult jassid as compared with Lambda cyhalothrin-based insecticides at all sites. The Tanzania Cotton Board has recently noted the effectiveness of Imidacloprid against cotton jassids, and the available report (TCB, 2025) recommends the insecticide as the product of choice for cotton growing areas of Tanzania that have been devastated by sucking pests.

The effectiveness of Imidacloprid-based insecticides are probably due to its mode of action and chemical properties, as it acts as a nicotinic acetylcholine receptor agonist, binding to insect-specific receptors and preventing acetylcholine breakdown (44–48). This causes continuous nerve stimulation and paralysis to death. Imidacloprid-based insecticides have also been reported to exhibit trans laminar activity that enables penetration of leaf tissue and acropetally movement (upward through the xylem), providing control of pests feeding on the underside of leaves after foliar application (49). The effectiveness of Imidacloprid-based insecticides against cotton jassids has previously been demonstrated, with mortality rates reaching between 63.49% and 92.42% upon application (50, 51).

Our findings are also supported by the study by Pate et al. (52) that Imidacloprid was very effective in controlling sucking insect pests including cotton jassid in cotton fields. Profenofos was the second most effective providing a potential of serving as an alternative to Imidacloprid in order to delay the development of resistance to insecticides in cotton pests. Casida and Durkin (45, 48) attributed its effectiveness to its mode of action whereby it blocks the enzyme acetylcholinesterase (AChE) through phosphorylation of a serine residue at the enzyme’s active site. This action prevents the breakdown of acetylcholine, resulting in its accumulation and leading to neurotoxic effects in both the central and peripheral nervous systems. It was further elucidated that some organophosphates require conversion into a more active oxon form (53, 54); the basis upon the relatively lesser effectiveness to Imidacloprid could be attributed. Nevertheless, the recorded effectiveness was regarded as adequate for jassid control.

Profenofos has also been reported to exhibit translaminar activity despite being described as a non-systemic insecticide with contact and stomach action against various pests. The limited effectiveness recorded with Lambda-cyhalothrin and Chlorpyrifos against jassids further reinforces the generally accepted facts that non-systemic insecticides may not be ideal to sucking pest dominated field circumstances. The two insecticides not have significant translaminar activity (53, 55), making them less ideal in managing sucking pests. Imidacloprid- and Profenofos-based insecticides are also effective against chewing insect pests that can cause substantial yield losses. Previous studies have reported their high efficacy in controlling pests such as the cotton bollworm in cotton crops by affecting their nerve cells, disrupting mitochondrial energy production, and inducing internal oxidative damage, which leads to stunted growth and abnormal development (56–58). The present study further highlights the superior performance of Imidacloprid and Profenofos in managing jassid infestations. Despite this, adoption among smallholder cotton growers remains limited due to several practical factors. The two products are relatively new and recently introduced into the Tanzanian cotton production sector. Many farmers continue to rely on older insecticides, particularly Chlorpyrifos, primarily because of its lower cost, ready availability, and long-term familiarity to farmers. Reliance on less effective products can increase pest pressure, necessitate more frequent applications, raise production costs, and accelerate the development of resistance. Distribution strategies, especially those managed by the Tanzania Cotton Board (TCB), may not fully align with current efficacy data, as Chlorpyrifos and Lambda-cyhalothrin continue to dominate supplies, likely reflecting procurement decisions mainly driven by costs more than insecticide performance.

An important consideration for the long-term sustainability of the chemical control of cotton jassids is the documented capacity of *Amrasca* species to develop resistance to multiple insecticide classes. Studies from Pakistan and India, where *A. biguttula* has been subjected to intensive insecticide pressure for decades, have reported high levels of resistance to neonicotinoids, including Imidacloprid (3.35-95.0-fold resistance ratios) and thiamethoxam (up to 1,198-fold) (36, 59). Similarly, resistance to organophosphates including Chlorpyrifos (2.65-36.42-fold) and Profenofos (7.28-57.71-fold) has been documented (37a). Pyrethroid resistance, including to Lambda-cyhalothrin, was first reported by Ahmad et al. (60) in Pakistani populations. The Arthropod Pesticide Resistance Database (61) lists multiple cases of *Amrasca* spp. resistance to all four insecticide classes tested in the present study. Although resistance levels in Tanzanian jassid populations have not yet been characterized, the relatively low efficacy of Chlorpyrifos and Lambda-cyhalothrin recorded in this study may indicate reduced susceptibility or possible early stages of resistance development. Proactive resistance monitoring through periodic bioassays should therefore be incorporated into IPM programs for cotton in Tanzania. Notably, newer chemistries such as flonicamid and sulfoxaflor have shown no resistance in *Amrasca* populations to date (37b) and could serve as future rotation partners.

While the present study focused on insecticide efficacy against jassids, the potential adverse effects of these chemicals on non-target organisms, human health, and the environment must be considered in any IPM strategy. Imidacloprid, although the most effective insecticide in this study, is highly toxic to pollinators such as honeybees (*Apis mellifera*), causing reduced survival, impaired foraging behavior, and reproductive effects at sublethal concentrations (62). Neonicotinoid applications in cotton have also been reported to reduce beneficial arthropod abundance by approximately 60%, particularly affecting *Chrysoperla* spp. (green lacewings) and coccinellids (ladybird beetles) that are important natural enemies of jassids (63, 64). Profenofos, classified as moderately hazardous (WHO Class II), has been associated with chromosomal aberrations, DNA damage, and reproductive toxicity in mammals (65) and reduces the feeding capacity of key predators such as *Cryptolaemus montrouzieri* and *Chrysoperla carnea* (66). Lambda-cyhalothrin is toxic to a wide range of natural enemies including spiders and predatory bugs at concentrations below recommended field application rates (67, 68). Of particular concern is chlorpyrifos, which has been banned in the European Union since 2020 and listed under the Stockholm Convention on Persistent Organic Pollutants in 2025 due to demonstrated neurotoxicity at doses below the threshold for cholinesterase inhibition, endocrine disruption, and environmental persistence (69–71). These findings underscore the need for judicious use of chemical insecticides within an IPM framework that incorporates biological control, host plant resistance, and cultural practices to minimize environmental impact.

The present findings also underscore the need for TCB and relevant stakeholders to review procurement and distribution policies, prioritizing on more effective insecticides and to consider the pesticide spectrum. It should be noted that Imidacloprid is highly effective against sucking pests, whereas Profenofos offers broader activity, including some chewing pests such as bollworms, although neither may provide complete, season-long protection when used alone.

Therefore, despite their effectiveness, these insecticides should be used as components of an IPM strategy that incorporates complementary chemistries and non-chemical approaches. Integrated pest management (IPM) for cotton jassids should also encompass multiple tactics beyond chemical control. Cultural practices include avoiding excessive nitrogen fertilization, planting at the right time, removing alternate host plants such as okra and *Cynodon dactylon* from field borders, and maintaining optimal plant populations (18). Host plant resistance is a promising strategy, as cotton cultivars with dense trichomes (leaf hairs) on the lower leaf surface significantly reduce jassid oviposition and feeding (13, 72–74). Biological control agents, including egg parasitoids (*Anagrus* spp., Mymaridae), predators such as green lacewings (*Chrysoperla carnea*), ladybird beetles (Coccinellidae), and spiders, contribute to natural suppression of jassid populations (75). Entomopathogenic fungi have recently been discovered attacking *A. biguttula* in Benin (76, 77), suggesting potential for bio-control development in Africa. Botanical pesticides, particularly neem-based formulations, have shown significant efficacy against jassids (78) and could complement synthetic insecticides in rotation programs. For Tanzania specifically, the Tanzania Cotton Board should consider promoting IPM packages that combine threshold-based insecticide applications using the most effective products identified in this study (Imidacloprid and Profenofos), rotated with alternative chemistries to delay resistance, alongside cultural practices and conservation of natural enemies.

Routine resistance monitoring could be implemented in Tanzania through periodic leaf-dip bioassays or topical application bioassays on field-collected jassid populations, comparing LC50 values against baseline susceptibility data. Such monitoring could be coordinated through the Tanzania Cotton Board in collaboration with research institutions such as TARI and the Sokoine University of Agriculture. While establishing a comprehensive resistance monitoring program requires an initial investment in laboratory infrastructure and trained personnel, simplified diagnostic-dose bioassays using discriminating concentrations could provide a cost-effective first-tier screening approach suitable for cotton field conditions (37, 60a). As resistance to all four tested insecticide classes has been documented in Amrasca populations elsewhere, establishing baseline susceptibility data for Tanzanian populations should be considered a priority. Cost remains a critical factor in farmers’ insecticide selection. Although Imidacloprid is typically more expensive per unit than Chlorpyrifos, its superior efficacy reduces the number of required spray applications, potentially lowering overall production costs. Extension programs should thus prioritize educating farmers on these cost–benefit dynamics, emphasizing yield gains and profitability. While a full cost–benefit analysis was beyond the scope of the present study, future research should integrate yield data and detailed economic assessments to provide evidence-based recommendations for resource-constrained smallholder cotton farmers.

## 5 Conclusions and recommendations

The study demonstrated that Imidacloprid-based insecticides are highly effective in controlling both adult and nymph cotton jassids under the experimental field conditions tested at all three study sites (Kilosa, Bunda, and Misungwi), achieving over 67% mortality within 72 h and maintaining populations below economic threshold levels. These results are promising and warrant validation in larger-scale field trials across cotton-growing areas of Tanzania. Profenofos-based insecticides also provided significant, albeit lower suppression and may be used in rotation to delay resistance. It is recommended that Imidacloprid should serve as the primary option for jassid management, with Profenofos applied strategically within Integrated Pest Management (IPM) programs. Sustainable control should include farmer training on correct choices of insecticides to use, appropriate application techniques, judicious rotation of insecticides, and routine monitoring for possible resistance. Future research should explore combined applications of Imidacloprid and Profenofos, assess long-term ecological impacts, and investigate environmentally friendly alternatives such as bio-pesticides to ensure effective, sustainable cotton production.

## Data Availability

The raw data supporting the conclusions of this article will be made available by the authors, without undue reservation.
