# Supplementary material for: Responses of cotton jassid (Amrasca biguttula) to synthetic insecticides recommended in Tanzania
Source: Front Insect Sci. 2026 Apr 20;6:1774983. doi: 10.3389/finsc.2026.1774983 (PMC13136150; doi:10.3389/finsc.2026.1774983)
Supplement: Supplementary file 1 [file DataSheet1.pdf]

SUPPLEMENTAL FILE FOR FIGURES AND TABLES

1. FIGURES

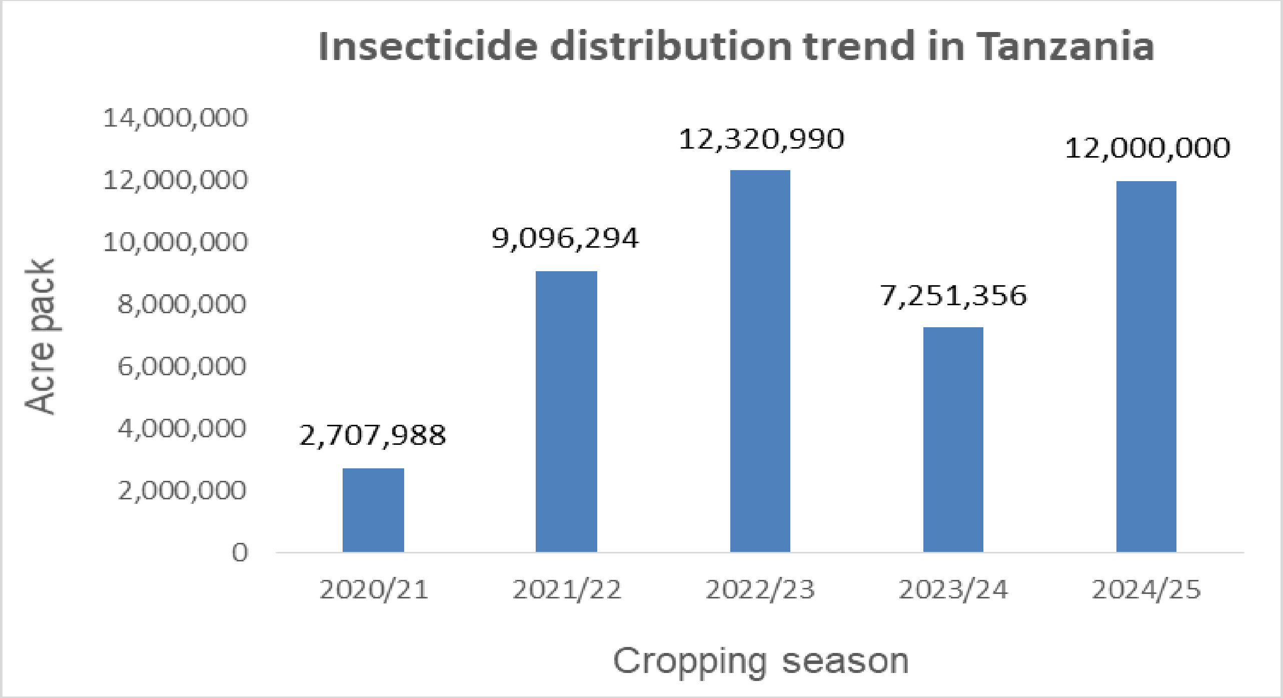

Fig S1: Insecticide use in the cotton sector during the 2020/21–2024/25 seasons. Source: TCB, 2025

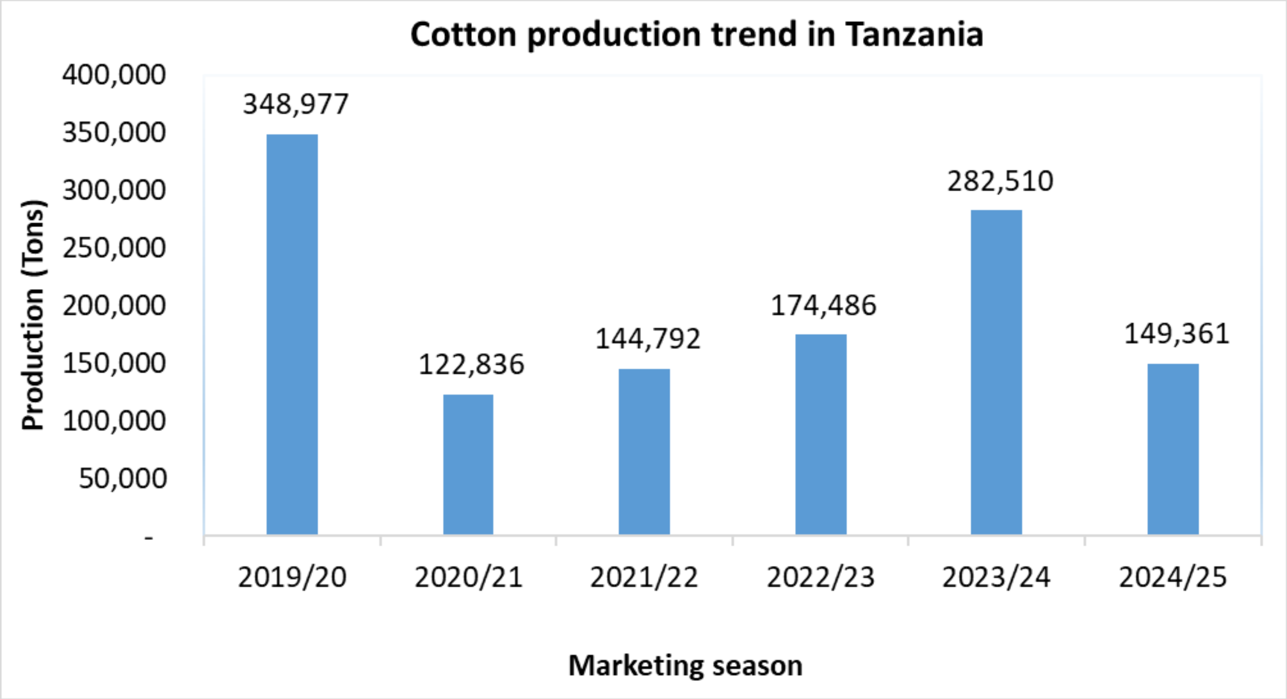

Fig S2: Cotton production trend from the 2019/20 to 2024/25 marketing season. Source: TCB, 2025.

2. TABLES

Table S3: Site × treatment interaction effects on adult cotton jassid mean populations (AV-MEAN) per plant at 24, 48, and 72 hours post-application across three spray rounds in Bunda, Kilosa, and Misungwi, Tanzania.

SITE*TRT – ADULT JASSIDS															
SITE	TRT	Before spray 1	Spray 1			Before spray 2	Spray 2			Before spray 3	Spray 3			AV - MEAN before three sprays	AV - MEAN after three sprays
			24hrs	48hrs	72hrs		24hrs	48hrs	72hrs		24hrs	48hrs	72hrs		
**Kilosa**	**T1**	10.00	4.00	2.33	1.67	7.00	5.00	2.00	1.33	6.00	2.67	2.00	1.67	7.67	2.52
**Bunda**	**T1**	4.00	2.33	1.33	1.33	3.33	2.33	2.00	1.33	9.33	4.00	2.67	2.00	5.55	2.15
**Misungwi**	**T1**	9.33	3.33	2.33	1.33	5.00	2.00	1.67	1.33	4.67	2.33	1.67	1.33	6.33	1.93
**Kilosa**	**T2**	9.00	7.00	5.00	3.33	12.00	10.00	6.67	7.33	12.67	8.00	8.00	5.00	11.22	6.70
**Bunda**	**T2**	6.67	5.00	3.67	4.00	5.00	4.00	3.00	4.00	18.33	10.00	10.33	7.67	10.00	5.74
**Misungwi**	**T2**	9.67	6.33	3.67	4.33	7.00	5.33	5.33	4.33	7.67	5.67	4.67	3.00	8.11	4.74
**Kilosa**	**T3**	10.67	9.67	6.33	4.67	12.33	12.00	9.33	11.33	14.00	11.33	10.00	7.67	12.33	9.15
**Bunda**	**T3**	5.33	4.67	4.00	5.33	5.33	4.67	4.67	6.00	17.33	14.33	13.00	9.00	9.33	7.30
**Misungwi**	**T3**	9.67	9.00	5.67	5.67	9.00	7.00	6.67	7.33	8.00	7.67	6.00	4.33	8.89	6.59
**Kilosa**	**T4**	9.33	6.00	4.00	2.67	9.33	7.67	5.00	3.33	13.67	6.33	6.67	3.67	10.78	5.04
**Bunda**	**T4**	6.00	3.33	3.00	3.00	4.00	3.33	3.00	2.00	22.67	11.67	8.33	5.67	10.89	4.81
**Misungwi**	**T4**	10.00	5.00	3.33	3.00	8.67	4.00	3.67	3.67	6.33	3.33	2.00	2.67	8.33	3.41
**Kilosa**	**Tc**	9.67	12.00	12.33	13.33	12.67	13.67	14.67	15.00	16.00	15.00	20.67	21.00	12.78	15.30
**Bunda**	**Tc**	12.00	13.00	10.67	13.00	8.00	8.33	9.33	10.33	15.67	20.00	19.33	22.33	11.89	14.04
**Misungwi**	**Tc**	9.33	11.00	12.33	13.67	11.33	12.67	10.67	13.00	12.67	13.00	11.67	14.00	11.11	12.44
**P-Value**		0.07	0.02	0.93	0.99	0.86	0.72	0.24	0.26	0.02	0.61	0.36	0.26		
**LSD**		3.77	2.18	2.13	2.24	4.39	4.30	2.79	3.15	4.78	4.54	4.87	4.03		
**c.v (%)**		25.80	19.30	23.80	25.00	32.80	37.80	28.50	30.90	23.20	30.10	34.40	32.50		

Note: Footnote: T1–T4 = Insecticide treatments (imidacloprid, lambda-cyhalothrin, chlorpyrifos, profenofos); Tc = untreated control. AV-MEAN refers to mean jassid nymph counts per plant. Data analyzed by ANOVA; P-values indicate significance of site × treatment interaction; LSD = least significant difference ($p < 0.05$); CV = coefficient of variation.

Table S4: Site × treatment interaction effects on nymph cotton jassid mean populations (AV-MEAN) per plant at 24, 48, and 72 hours post-application across three spray rounds in Bunda, Kilosa, and Misungwi, Tanzania.

SITE*TRT – NYMPH JASSIDS															
**SITE**	**TRT**	**Before spray 1**	**Spray 1**			**Before spray 2**	**Spray 2**			**Before spray 3**	**Spray 3**			**AV - MEAN before three sprays**	**AV - MEAN after three sprays**
			**24hrs**	**48hrs**	**72hrs**		**24hrs**	**48hrs**	**72hrs**		**24hrs**	**48hrs**	**72hrs**		
**Bunda**	T1	10.67	4.00	1.67	1.33	5.67	3.33	2.33	2.33	11.33	6.67	3.00	2.33	9.22	3.00
**Kilosa**	T1	8.33	4.33	2.33	1.67	8.00	5.00	2.33	1.67	2.67	1.67	1.33	1.00	6.33	2.37
**Misungwi**	T1	14.00	4.00	2.00	1.33	6.00	3.00	2.67	2.33	4.67	2.67	1.67	1.33	8.22	2.33
**Bunda**	T2	8.00	6.33	6.00	5.33	7.00	5.67	5.67	4.67	22.33	20.67	19.00	16.33	12.44	9.96
**Kilosa**	T2	9.67	7.33	6.00	4.67	13.67	13.33	10.33	9.00	10.00	7.00	7.33	7.00	11.11	8.00
**Misungwi**	T2	11.00	8.67	9.00	6.33	9.33	6.00	5.00	5.33	8.33	6.67	6.00	4.67	9.55	6.41
**Bunda**	T3	7.67	4.67	5.67	4.00	7.33	5.33	4.33	3.67	23.00	16.33	13.33	13.33	12.67	7.85
**Kilosa**	T3	9.00	5.00	4.67	3.00	13.67	9.33	7.00	6.00	11.67	5.33	6.00	4.00	11.45	5.59
**Misungwi**	T3	8.33	7.33	8.00	4.67	9.33	6.00	4.00	4.33	8.00	5.00	3.33	3.00	8.56	5.07
**Bunda**	T4	8.67	5.33	2.67	2.00	6.33	5.00	3.67	3.33	22.67	14.00	11.00	10.33	12.56	6.37
**Kilosa**	T4	10.33	6.67	3.67	2.67	13.67	6.33	4.67	3.67	6.00	3.33	5.00	3.00	10.00	4.33
**Misungwi**	T4	10.00	6.33	4.67	3.00	8.67	5.33	3.33	3.33	6.33	4.33	2.33	2.33	8.33	3.89
**Bunda**	Tc	10.67	11.33	10.00	11.67	10.67	11.67	12.00	10.00	28.33	28.67	29.67	30.33	16.55	17.26
**Kilosa**	Tc	8.33	9.33	9.67	12.00	19.33	16.00	18.33	19.00	12.00	11.00	12.67	14.00	13.22	13.56
**Misungwi**	Tc	8.33	10.67	10.67	11.33	11.00	11.33	13.00	14.33	10.33	11.00	11.67	12.00	9.89	11.78
**P-Value**		3.80	0.50	0.30	0.43	0.43	0.43	0.28	0.01	0.25	0.25	0.01	<.001		
**LSD**		0.19	2.77	2.18	1.68	3.99	3.99	3.79	3.15	6.00	6.00	5.39	3.48		
**c.v (%)**		23.80	24.50	22.50	20.10	23.90	23.90	34.40	30.40	28.70	28.70	36.30	24.90		

Note: T1–T4 = Insecticide treatments (imidacloprid, lambda-cyhalothrin, chlorpyrifos, profenofos); Tc = untreated control. AV-MEAN refers to mean jassid nymph counts per plant. Data analyzed by ANOVA; P-values indicate significance of site × treatment interaction; LSD = least significant difference ($p < 0.05$); CV = coefficient of variation.
